# Effect of a live attenuated vaccine against *Lawsonia intracellularis* in weaned and finishing pig settings in Finland

**DOI:** 10.1186/s13028-018-0374-8

**Published:** 2018-03-23

**Authors:** Kati Susanna Peiponen, Birger Taneli Tirkkonen, Jouni Juho Tapio Junnila, Mari Leena Heinonen

**Affiliations:** 1 Vetcare Finland Ltd, Hiomotie 3 A, 00380 Helsinki, Finland; 2 Atria Pork/A-Farmers Ltd, Po. Box 910, 60061 Atria, Finland; 3 Department of Production Animal Medicine, Faculty of Veterinary Medicine, University of Helsinki, Paroninkuja 20, 04920 Saarentaus, Finland; 4 4Pharma Ltd, Arkadiankatu 7, 00100 Helsinki, Finland

**Keywords:** Average daily weight gain, *Lawsonia intracellularis*, Live attenuated vaccine, Pig, Vaccination

## Abstract

**Background:**

The intracellular bacterium *Lawsonia intracellularis* is an important pathogen in modern swine production. The aim of this study was to investigate the effect of a live attenuated *L. intracellularis* vaccine (Enterisol Ileitis^®^) on the health and production parameters of weaned and finishing pigs in a commercial Finnish 850-sow farm with diagnosed *L. intracellularis* infection. The herd was free from enzootic pneumonia, swine dysentery, progressive atrophic rhinitis, sarcoptic mange and salmonellosis. Four weekly groups of approximately 500 piglets were included in the study for a total of approximately 2000 piglets. Half of these piglets were vaccinated at 3 weeks of age and the other half served as controls. The study piglets were ear-tagged with individual numbers and colour-coded and were individually weighed at weaning (4 weeks), delivery to the finishing farm (12–14 weeks) and at slaughter. Mortality, symptoms of diseases and medications of the study piglets were registered in the nursery and finishing unit. Feed conversion rate was calculated for the finishing period and lean meat percentage was measured at slaughter.

**Results:**

Vaccinated piglets had a higher live weight than unvaccinated piglets at delivery to the finishing unit (+ 1.18 kg, P = 0.002) and at slaughter (+ 3.57 kg, P < 0.001). The daily weight gain of vaccinated piglets was better than unvaccinated piglets in the nursery (+ 14.8 g/d, P = 0.013) and in the finishing unit (+ 30.9 g/d, P < 0.001). Vaccination had no effect on feed conversion rate or lean meat percentage (P = 0.102). Altogether, 3.9 and 4.6% of the pigs were medicated for different reasons in the vaccinated and control groups, respectively. The return on investment for the vaccination was calculated to be 0.41.

**Conclusions:**

Immunisation of piglets with a live attenuated *L. intracellularis* vaccine resulted in higher meat yield in pig production via significantly higher live weight and average daily weight gain in a Finnish specific pathogen-free setting.

## Background

The intracellular bacterium *Lawsonia intracellularis* is an important pathogen in modern commercial swine production. A disease with suspected infectious aetiology later known to be caused by *L. intracellularis* was first described in pigs in 1931 [1]. The disease is now recognised to have an economic impact via performance decrease in piglets, which in 2006 resulted in losses of approximately 1.5–3.0 USD per animal in the weaning period [2]. The costs arise from increased mortality, reduced daily weight gain and need for medications.

*Lawsonia intracellularis* is easily transmitted and is widespread in many countries, including the Nordic countries. The prevalence of *L. intracellularis* is 48% of piglet-producing herds in Sweden [3] and 93.7% of such herds in Denmark [4]. No reports have been published regarding the prevalence of *L. intracellularis* in Finland. There is anecdotal evidence that *L. intracellularis* is also a common pathogen causing enteric disease in Finnish pig herds [5].

*Lawsonia intracellularis* causes the condition known as proliferative enteropathy. This condition may lead to various symptoms, such as diarrhoea and wasting in growing pigs to acute intestinal haemorrhage in adult pigs naive to the pathogen [6]. Diagnosis is made via laboratory identification of antibodies against *L. intracellularis* from blood samples, direct identification of bacteria or bacterial DNA from faecal samples or by identification of typical morphological changes for *L. intracellularis* infection at necropsy. Many antimicrobial agents can be used for the treatment of proliferative enteropathy. In Finland, the Finnish Food Safety Authority Evira recommends tylosin or other macrolides (first-line treatment) or tiamulin or tetracycline (second-line treatment) [7]. Although antimicrobial use in Finnish pork production is among the lowest worldwide [8], Finland is aiming to decrease this use further. One way to lower medication use for intestinal infections is to identify alternative means to control *L. intracellularis* in pig farms. Vaccination is investigated as one possible measure and is also mentioned in the national recommendations.

Vaccination against *L. intracellularis* has been previously reported in some successful field studies in Denmark [9], Australia [10], Korea [11], Switzerland [12] and Hungary [13]. These studies reported increased weight gain, decreased mortality and lower antimicrobial use. Some studies have shown that vaccination increased the immunity of piglets against *L. intracellularis* and decreased intestinal lesions and bacterial shedding [14–17]. In Finland, the pathogen load of herds is different from those mentioned above; most of the herds belong to the health classification system Sikava that requires absence of enzootic pneumonia, swine dysentery, salmonella, progressive atrophic rhinitis and sarcoptic mange [18]. The results of *L. intracellularis* vaccine use have not been reported in herds with a similar specific pathogen-free (SPF) system.

This study aimed to investigate the effect of a live attenuated *L. intracellularis* vaccine in a Finnish sow farm with diagnosed *L. intracellularis* infection. The goal of the study was to measure the effect of the vaccine when used in a Finnish SPF setting. Our first hypothesis was that *L. intracellularis* vaccination would have a positive effect on production, namely better weight gain, lower mortality and morbidity, smaller variation in slaughter weight, better feed conversion rate and financially better return on investment. We also hypothesised that *L. intracellularis* vaccination use is associated with lower antimicrobial use.

## Methods

### Procedures in the study herds

The study was performed in a piglet-producing farm with 850 sows and in a finishing herd with room for 1632 finishing pigs. Both herds were free from *Salmonella* sp., *Mycoplasma hyopneumoniae*, *Brachyspira hyodysenteriae*, sarcoptic mange and progressive atrophic rhinitis. The sow farm had a history of persistent diarrhoea typical of *L. intracellularis* in nursery pigs 2–3 weeks after weaning. The herd was diagnosed with a *L. intracellularis* infection in March 2014. Four piglets with typical diarrhoea were euthanised and sent for necropsy at Finnish Food Safety Authority Evira. One of the piglets was diagnosed with *L. intracellularis* with macroscopic and histologic lesions typical of proliferative enteropathy and identification of *L. intracellularis*-like organisms in Warthin–Starry stained tissue sections. Blood samples were obtained from six (age 6 weeks) and 11 (age 12 weeks) piglets; one (17%) and 11 (100%), respectively, were serologically positive in an ELISA test for *L. intracellularis*. Two finishing pigs from a farm receiving piglets only from the study sow farm were also investigated at Evira. Necropsy findings revealed *L. intracellularis*-infection in both of the finishers. The diagnosis was confirmed by examination of Warthin–Starry stained sections (one case) and by PCR (EVIRA6307 PCR for *Lawsonia intracellularis*) (one case). The farm had started to treat the pigs prophylactically with oral tylvalosin, 4.25 mg/kg (Aivlosin^®^, ECO Animal Health Limited) for 10 days from 3 days after weaning.

The sow farm weaned approximately 500 piglets every week in one of their all-in-all-out nursery departments. At weaning, the piglets were on average 28 days old. About 15% of the piglets were too small to be weaned at 4 weeks; their weaning was postponed until they were 5 weeks old. Litters from most sows were mixed at weaning, but the vaccine and control groups were raised in their own pens.

The nursery pen floor consisted of one-third concrete slats and two-thirds of solid concrete floor with floor heating. Every pen had a liquid feeding system with long troughs and two water nipples. Altogether, 23–26 piglets were placed in every pen, providing them with approximately 0.35 m^2^ of floor space per piglet.

After a rearing time of approximately 7 weeks in the nursery, piglets in the trial group were delivered to a finishing farm, where also their feed consumption could be followed. The finishing period of the piglets moved to two other farms were not included in the study.

Piglets were delivered to the finishing farm in 4 weekly groups of 408 piglets; half of were vaccinated and half served as controls (total 1632 piglets). All 408 pigs of the same batch were in one compartment and no other pigs were kept in the same compartments. Vaccinated and control pigs were kept in separate pens throughout the study and they were not mixed with pigs not included in this study.

The finishing farm had four compartments with 34 pens per compartment and 12 pigs per pen, providing about 0.9 m^2^ for each pig. Two pens shared one feed distribution valve. The pigs were fed five times a day with a liquid feeding system with approximately 32 cm of trough per pig.

All pigs in one trial group (originally 408 pigs) were sent to slaughter on the same day even if all of them had not reached the optimum slaughter weight. Due to higher than normal stocking density in the pens, eight pigs had to be culled before slaughter and were excluded from the study. Under normal production circumstances, the farmer would have sent these pigs to slaughter in two or three batches as they reached the optimal slaughter weight, thus lowering the stocking density for the last days of final production phase. The delivery date to slaughter was fixed by batches in the study so that the pigs could be followed at the slaughterhouse. The pigs in this study were sent to slaughter 79–86 days after arrival in the finishing farm. They were tattooed on their backs the day before transport to slaughterhouse. The tattoo included the following information: farm identity number, compartment number and treatment group (V = vaccinated and C = control group). Most of the slaughter pigs also had their individual identification ear tags intact at the time of transport to slaughter.

### Vaccination and control groups

Four weekly weaning groups were followed in the study. One group consisted of approximately 500 piglets, of which half were vaccinated with *L. intracellularis* vaccine at the age of 3 weeks about 7 days before weaning (V = vaccine group). The other half of non-vaccinated piglets served as controls (C = control). The litters were chosen randomly and all piglets in a litter were either vaccinated or served as controls. One vaccine group piglet at a time was lifted from the ground and administered 2 mL of *L. intracellularis* vaccine (Enterisol Ileitis^®^, Boehringer Ingelheim Vetmedica GmbH) directly into the mouth with a drench and injected intramuscularly 1 mL of circovirus vaccine (Ingelvac CircoFLEX^®^, Boehringer Ingelheim Vetmedica GmbH) behind its ear. The control group piglets were lifted similarly but only received the circovirus vaccine. Vaccinated piglets were marked with an individually numbered red ear tag in their right ear. Similarly, control piglets received a numbered yellow ear tag in their left ear.

### Follow up of weight and weight gain

All study piglets were weighed individually at weaning (weaning weight) and within a week prior to delivery to the finishing farm (delivery weight). At that time, they were roughly 30 kg live weight and 12–14 weeks of age.

The slaughterhouse provided the individual carcass weight of each pig. Live weight at slaughter was calculated from the carcass weight by conversion from 74.5% slaughter yield. Unfortunately, the ear tags for roughly half of the pigs (n = 807) were lost during the scalding of the carcasses. This was likely due to the ‘widening’ of the ear tag hole during the growth of the pinnae, which caused the tag to fall off easily. Therefore, we could obtain a partial data set with individual parameters from some of the pigs and the data with parameters with group-level accuracy from the pigs that lost their ear tag.

### Medications and treatments of the pigs

Routine or group treatment antimicrobials were not used for the trial groups. Because of the use of a live vaccine, we advised the farmer not to give any antimicrobial medications to piglets for 3 days before and after vaccination. Piglets that required medication with antimicrobials during these restriction days were excluded from the trial.

The animals were medicated due to sickness or injury according to the normal treatment routine advised by the herd veterinarian. The farmer recorded all medications for the piglets both in the nursery and in the finishing units individually, including date, indication or symptoms and medicine used.

### Mortality

The farmers both in the nursery and in the finishing unit registered individually the dates of death and euthanasia.

### Feed conversion rate

Feed consumption was registered at the pen level by the finishing farmer for different study groups in a table planned for this purpose. As vaccination and control piglets were kept in separate pens, feed consumption could be then traced at the vaccination versus control level.

### Lean meat percentage

The slaughterhouse provided the information of the lean meat percentage for each pig individually at slaughter; this was measured with the AutoFom system (Carometec A/S, Denmark).

### Return of investment

The return on investment (ROI) for the vaccine was calculated as

$$ \left( {increased \,revenues \left( {increased \,meat\, from\, the \,slaughterhouse} \right)\, - \,increased \,costs \left( {price\, of\, vaccine\, + \,price \,of\, labour\, + \,increased \,feed \,intake \,for\, increased \,live\, weight} \right)} \right)\, \div \,increased \,costs. $$

### Statistical methods

Weight at weaning was considered as the baseline measurement. The difference between treatment groups in change from baseline in weight was analysed with repeated measures analysis of covariance model (RM-ANCOVA). The model included vaccination group, timepoint and the interaction between treatment group and baseline measurement as fixed terms, the baseline measurement as covariate and the pig as a random term. The difference between the treatments overall and at delivery and slaughter was estimated from the model using contrasts. A similar RM-ANCOVA model was fitted for the response of daily growth (g). Daily growth was defined in two different ways. First, the daily growth was calculated from the previous measurement. Second, the daily growth was calculated from the baseline measurement, regardless of the timepoint as a sensitivity analysis.

Weight was also analysed using the actual values (instead of change from baseline) by applying a linear mixed model for repeated measures. The model included treatment, timepoint and their interaction as fixed terms and the pig as a random term.

For the pigs missing their ear tags at scalding, daily growth could not be calculated (as the values of the baseline and delivery measurement were unknown). To analyse the full data at slaughter weighing, the following data imputations were performed. The baseline (weaning) date was imputed as the first date of the two possible weighing dates within the group. The first date was selected as a vast majority of the pigs (80–90%) were weighed on the first date. The delivery date was imputed as the date of delivery weighing of the corresponding group. The baseline/delivery weight of the pigs with missing ID at slaughter was imputed as the mean weight of the corresponding group and treatment.

Using the imputed data, an analysis of covariance (ANCOVA) model was fitted for the daily growth at slaughter. The model included the treatment group and the baseline-weight covariate as fixed terms.

The incidence of deaths between the treatment groups separately at both delivery and slaughter was compared with Fisher’s exact tests.

The meat percentage between the treatment groups at slaughter was analysed with independent samples t-test.

The feed consumption was reported by pen in the original data (without pig identifications) and all calculations were made at the pen level. To calculate the feed conversion ratio, the following decisions on calculations and data derivations were made to make the values as unbiased as possible:

- The total feed consumption of the pen was calculated as:
  $$ feed\, consumption \left( {energy} \right)\, \times \,number\, of\, pigs\, in\, the\, pen. $$
  Deceased and euthanised pigs were removed from the total number of pigs in the pen.
- To calculate the overall weights at delivery and slaughter, the weight data was modified as follows: pigs reported as deceased or euthanised were deleted from the data. Using this dataset, the total weight at delivery and slaughter by treatment and trial group were calculated.
- The feed conversion ratio at the pen level was calculated with the following formula:
  $$ {\text{feed consumption}}\, \div \,\left( {{\text{weight at slaughter}}\, - \,{\text{weight at delivery}}} \right). $$

All statistical analyses were performed at 4Pharma Ltd using SAS^®^ System for Windows, version 9.3 (SAS Institute Inc., Cary, NC, USA).

## Results

### Weight and daily weight gain

Vaccinated pigs were significantly heavier than control pigs at time of delivery to the finishing unit and at slaughter. Daily weight gain was better in vaccinated pigs than that in control pigs. Detailed results of the weights and average daily weight gains are presented in Tables 1 and 2. Live pig weight data was obtained from the individual weighings of identified pigs. The weight data from the slaughtering process was identified only at the treatment group level (vaccine vs control).

**Table 1:** Weight of the study pigs at different timepoints during the study

Timepoint	Vaccinated pigs		Control pigs		Mean difference, kg (SE)*	P-value*
	n	Weight in kg, average (SD)^#^	n	Weight in kg, average (SD)^#^		
Weaning	962	7.1 (1.2)	960	6.9 (1.2)	0.1 (0.4)	0.697
Delivery to finishing unit	861	24.1 (7.0)	923	22.9 (6.9)	1.2 (0.4)	0.002
Slaughter	800	103.9 (13.8)	800	100.3 (12.3)	3.6 (0.4)	< 0.001

Vaccinated piglets received an oral dose of *L. intracellularis* vaccine at the age of 3 weeks; control pigs were untreated

*n* number of pigs

^#^Descriptive data, * based on the linear mixed model for weight

**Table 2:** The average daily weight gain of the study pigs at different timepoints during the study

Timepoint	Vaccine		Control		Mean difference (SE)*	P-value*
	n	Average daily weight gain g/day (SD)	n	Average daily weight gain g/day (SD)		
Weaning-delivery	842	325.1 (123.1)^#^	905	306.0 (123.3)^#^	14.8 (6.0)	0.013
Delivery-slaughter	800	938.6^§^	800	907.7^§^	30.9 (7.4)^§^	< 0.001

Vaccinated piglets received an oral dose of *L. intracellularis* vaccine at the age of 2 weeks; control pigs were untreated

*n* number of pigs

^#^Descriptive data, * based on the statistical models, ^§^ imputed data

### Mortality

In the nursery, 64 vaccinated (6.5%) and 55 control piglets (5.6%) died or were euthanised (P = 0.451). In the finishing farm, nine pigs (1.2%) in both groups were found dead or were euthanised due to sickness. There was no statistical difference between the groups.

### Medications and clinical symptoms

At the time of the study, the nursery farmer decided not to use any medications in the nursery; the researchers had no influence on this decision. Instead, the farmer decided to euthanise all piglets in need of treatment for economic reasons. These piglets are included in the mortality figures described above.

The medications used in the finishing farm were minimal and the medications consisted of 68 individually treated pigs. A total of 3.9 and 4.6% of the vaccinated and control pigs were medicated during finishing, respectively. Treatments included injectable and peroral antimicrobials, nonsteroidal anti-inflammatory agents or trace minerals. The clinical symptoms recorded with medications that could be linked to *L. intracellularis* were diarrhoea (4 and 6 pigs in the vaccination and control groups, respectively), rectal prolapse (2 pigs in the vaccination group) and anorexia (0 in the vaccination group, 1 in control group). Altogether, 55 pigs were treated in the finishing unit due to symptoms unlikely to be related to intestinal disorders (lameness and inability to stand up, arthritis, abscesses, tail biting, and runts).

### Feed conversion ratio

The mean feed conversion ratio (FCR) in the finishing unit was 2.63 FE/kg (vaccination group) and 2.65 FE/kg (control group).

### Lean meat percentage

For lean meat percentage, a total of 1600 pigs were included in the analysis (800 vaccinated pigs and 800 control pigs). The mean lean meat percentage for vaccinated pigs was 59.3% (SD 3.3) and 59.6% (SD 3.8) for control pigs. There was no statistical difference between the groups (P = 0.102).

### Return on investment

During the study, the price per kilogram of meat paid to the farmer from the slaughterhouse was 1.40 EUR. As the difference between vaccination and control group in live weight was 3.6 kg at slaughter, the estimated difference in kg meat was 2.7 kg (3.6 * 0.745). The increased revenue was thus 3.78 EUR per pig. The costs were estimated to be 0.70 EUR per piglet in the Finnish setting, including the costs of the vaccine and labour. The cost of increased feed intake was also included in the calculation and was estimated to be 1.99 EUR (2.63 FE/kg * 3.6 kg * 0.21 EUR/FE).

Return of investment was calculated to be with these estimates 0.41.

## Discussion

Our results indicate that oral vaccination with live attenuated *L. intracellularis* resulted in both a significantly higher live weight and a significantly higher average daily weight gain (ADWG) than that of the control group. This is consistent with other results from vaccination field trials [11]. This indicates that *L. intracellularis* is an important pathogen in a Finnish SPF setting at the herd level and can limit optimal performance throughout the chain. There was a need to evaluate the effect of the vaccine in an environment where the pathogen load is low compared to other similar trials.

The FCR was similar in treatment and control groups as seen in other published field trials [10]. The results might have been different if more accurate data regarding feed consumption was available. As the vaccine is reported to increase ADWG and decrease the formation of intestinal lesions in pigs [14], we can assume that this would also influence the FCR favourably.

There was no statistical difference in lean meat percentage between the groups. This indicates that vaccination leads via significantly higher slaughter weight to better gain in the amount of lean meat (kg) per slaughtered animal and is therefore a possible tool to increase the efficacy of pork production at the farm level. To the authors’ knowledge, this is the first time that the effect of *L. intracellularis* vaccination on lean meat percentage has been investigated in a field trial. In the study of McOrist et al. [10], back fat depth was reduced in vaccinated pigs, indicating ‘leaner’ growth patterns during the growing phase.

A decrease in antimicrobial usage after immunisation against *L. intracellularis* has been demonstrated in previous studies [9] and was also the hypothesis of our study. We could not confirm this difference. A possible explanation could be our more restrictive approach to oral group antimicrobial treatments and our relatively small sample size compared to the previous study by Bak and Rathkjen [9], where the sample size was altogether 15,656 pigs. Minimal antimicrobial use was attempted in our study herds, in accordance with the National Recommendations for the Use of Antimicrobials Against the Most Important Infectious Diseases of Animal [7]. Therefore, the starting level of antimicrobial use was already low, and a decrease was difficult to achieve. The obvious advantage with this study was that the farm could stop using the in-feed antimicrobial agents against *L. intracellularis* required for the weaned piglets before the vaccination trial started. We hypothesised that we would record more clinical symptoms typical of *L. intracellularis* in the control group, but this was not observed during the study. The disease of younger pigs has typically low mortality and most of the affected animals recover and thereafter grow normally [6]. It is possible that the overall reduced infection pressure and good management kept the clinical symptoms at a manageable level. However, as the significant difference in growth between the vaccinated and control pigs shows, infection in the control pigs had some effect even if the symptoms were subclinical.

The mortality during the nursery phase was much higher than that in other Finnish SPF farms. Statistics from the National Finnish Porcine Healthcare reports (2015 and 2016) reported national nursery mortality to be at the level of 1.6% and euthanised 0.7% of the piglets [19]. The owner decided to euthanise all sick animals in lieu of medication use. This was an unusual decision and we did not have access to data on the percentages of euthanised and dead pigs. This data would have provided a better understanding of the situation at farm level. In the finishing unit, the mortality was about the same as in most similar herds. Other *L. intracellularis* vaccination studies report similar mortalities in the vaccination and control groups [10, 11] or a lower mortality in the vaccination group [10, 12]. The effect of the vaccine can be different at the farm level as shown in McOrist et al. [10], which refers to the variable expression of the infection on different farms.

A positive ROI for the use of the vaccine was expected in this study, as it is demonstrated in previous studies [11, 12]. For example, Park et al. [11] estimated in their cost–benefit analysis a 4.2:1 ROI. However, the assumptions and circumstances (meat and feed price, pig health status) varied between the studies and generalisations on expected revenues between farms should be viewed with caution. As the mortality during finishing was low and similar between treatment groups and there were no differences in medication demand between groups, the ROI was based solely on increased revenues from increased meat production. The ROI is expected to be better in farms where mortality is more commonly observed due to *L. intracellularis* infection and where medications are needed.

There were some limitations to the study. It was challenging to follow all animals in a field setting and some of them were lost during the study period. The loss of ear tags during scalding also decreased the number of animals with individual numbering throughout the study. For future studies, it is advisable to plan the feed consumption measurements to a more precise level, as the method used in this study did not yield data that could be used in statistical calculations. As the study was conducted only on one sow farm and one finishing farm, the results should be interpreted with caution and may not be valid for the entire Finnish swine industry.

## Conclusions

Immunisation of piglets with live attenuated *L. intracellularis* vaccine resulted in a higher meat yield in pig production via significantly higher live weight and average daily weight gain in a Finnish SPF farm. Immunisation ultimately led to better financial results. Vaccination is a tool to improve production in farms diagnosed with *L. intracellularis* infection.
